# The ER Protein Translocation Channel Subunit Sbh1 Controls Virulence of Cryptococcus neoformans

**DOI:** 10.1128/mbio.03384-22

**Published:** 2023-02-07

**Authors:** Felipe H. Santiago-Tirado, Thomas Hurtaux, Jennifer Geddes-McAlister, Duy Nguyen, Volkhard Helms, Tamara L. Doering, Karin Römisch

**Affiliations:** a Department of Molecular Microbiology, Washington University Medical School, St. Louis, Missouri, USA; b Department of Molecular and Cellular Biology, University of Guelph, Ontario, Canada; c Center for Bioinformatics, Faculty of Natural Sciences and Technology, Saarland University, Saarbruecken, Germany; d Department of Biology, Faculty of Natural Sciences and Technology, Saarland University, Saarbruecken, Germany; University of Melbourne

**Keywords:** *Cryptococcus neoformans*, *Saccharomyces cerevisiae*, Sbh1, cell wall, protein translocation, virulence

## Abstract

The fungal pathogen Cryptococcus neoformans is distinguished by a cell-wall-anchored polysaccharide capsule that is critical for virulence. Biogenesis of both cell wall and capsule relies on the secretory pathway. Protein secretion begins with polypeptide translocation across the endoplasmic reticulum (ER) membrane through a highly conserved channel formed by three proteins: Sec61, Sbh1, and Sss1. Sbh1, the most divergent, contains multiple phosphorylation sites, which may allow it to regulate entry into the secretory pathway in a species- and protein-specific manner. Absence of *SBH1* causes a cell-wall defect in both Saccharomyces cerevisiae and C. neoformans, although other phenotypes differ. Notably, proteomic analysis showed that when cryptococci are grown in conditions that mimic aspects of the mammalian host environment (tissue culture medium, 37°C, 5% CO_2_), a set of secretory and transmembrane proteins is upregulated in wild-type, but not in *Δsbh1* mutant cells. The Sbh1-dependent proteins show specific features of their ER targeting sequences that likely cause them to transit less efficiently into the secretory pathway. Many also act in cell-wall biogenesis, while several are known virulence factors. Consistent with these observations, the C. neoformans *Δsbh1* mutant is avirulent in a mouse infection model. We conclude that, in the context of conditions encountered during infection, Sbh1 controls the entry of virulence factors into the secretory pathway of C. neoformans, and thereby regulates fungal pathogenicity.

## INTRODUCTION

Cryptococcus neoformans is a haploid budding yeast that is ubiquitous in the environment, so that spores or desiccated cells are frequently inhaled (1, 2). In healthy individuals, these infectious particles are generally cleared or establish an asymptomatic latent infection (3). If, however, the host is or becomes severely immunocompromised, the pathogen may grow and disseminate, both within and outside host cells (4). Dissemination to the central nervous system results in a devastating meningitis, which causes almost 200,000 deaths each year worldwide (5).

Like other fungi, C. neoformans is protected by a multilayer cell wall, which is composed of a meshwork of chitin, chitosan, alpha and beta glucans, and mannoproteins (6). This flexible and dynamic structure responds to and protects the cell from environmental stresses, while accommodating morphogenesis. An additional role of the cryptococcal wall is to anchor an elaborate polysaccharide capsule that surrounds the cell (7, 8). This structure, which is unique among fungal pathogens, is required for cryptococcal virulence (1). Capsule polysaccharides are also shed from the cell into the surrounding environment, where they act to perturb the host immune response (9, 10).

**FIG 1:**
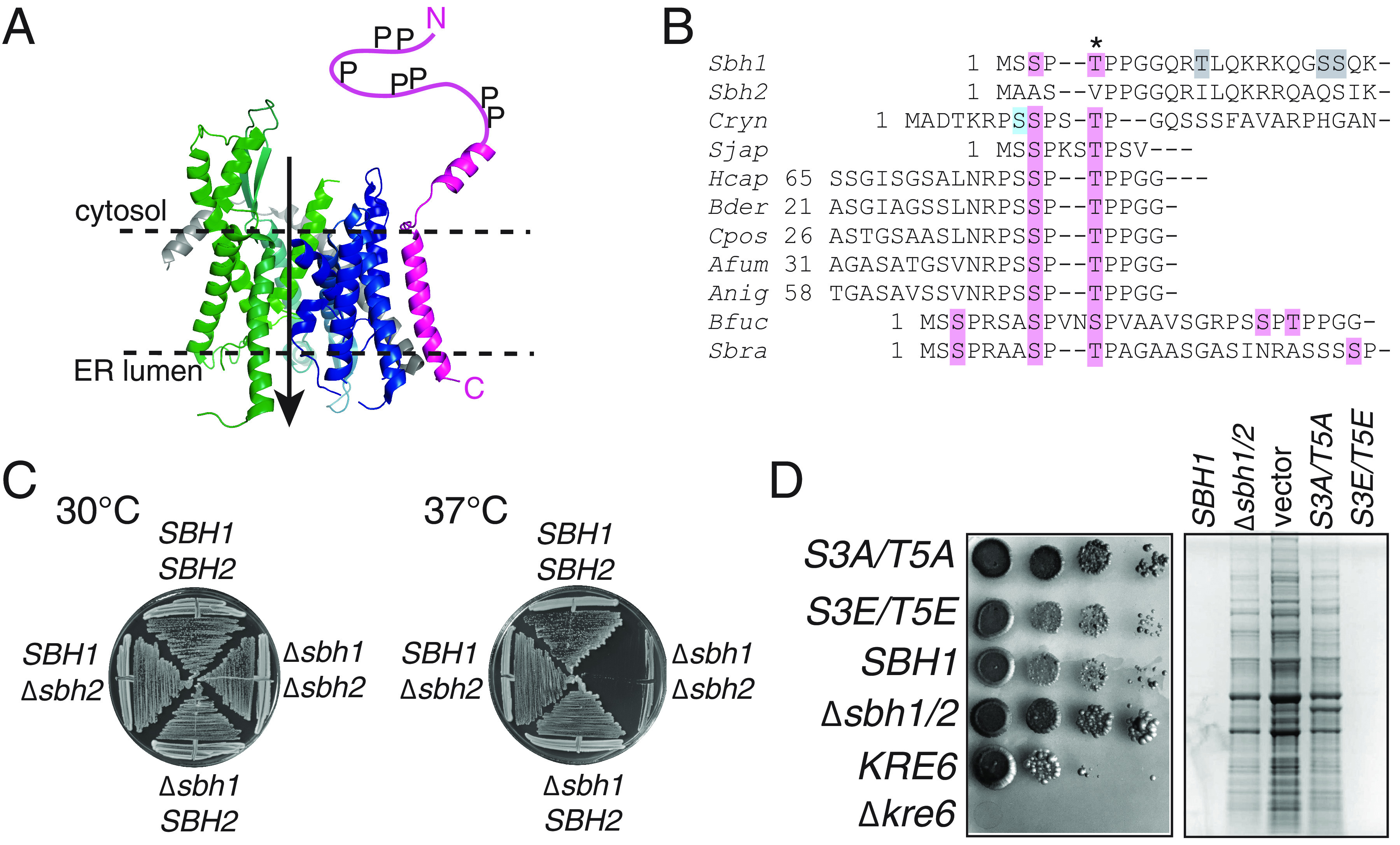
Sbh1 is required for cell wall integrity in S. cerevisiae. (A) Structure of the Sec61 channel of S. cerevisiae (modified from ref. 12; PDB 6ND1). The N-terminal half of Sec61 is shown in blue, C-terminal half in green, Sss1 in gray, and Sbh1 in magenta. The intrinsically unstructured N-terminal 38 amino acids of Sbh1 that were not visible in the cryo-EM structure were drawn in by hand. P, phosphorylation sites; dashed lines, position of the ER membrane; arrow, direction of protein transport. (B) Alignment of the poorly conserved N termini of Sbh1 proteins from S. cerevisiae (Sbh1, Sbh2) and the following pathogenic fungi: Cryn Cryptococcus neoformans, Sjap *Schizosaccharomyces japonicus*, Hcap Histoplasma capsulatum, Bder Blastomyces dermatitidis, Cpos *Coccidioides posadasii*, Afum Aspergillus fumigatus, Anig Aspergillus niger, Bfuc *Botryotinia fuckeliana*, Sbra *Sporothrix brasiliensis*. Except for Sbh2, only regions containing S or T residues with flanking prolines (pink) are shown. The conserved proline-flanked T5 in S. cerevisiae Sbh1 is indicated by an asterisk; other confirmed phosphorylation sites in S. cerevisiae Sbh1 are shown in gray; and the PKA site in C. neoformans Sbh1 is in blue. *Schizosaccharomyces cryophilus*, *Schizosaccharomyces octoporus*, Candida albicans, Schizosaccharomyces pombe, Kluyveromyces lactis, Yarrowia lipolytica, Pichia pastoris, and *Hansenula polymorpha* Sbh1 proteins were also screened, but do not contained potential proline-flanked phosphorylation sites. For full alignments of all Sbh1 protein sequences see Fig. S1. (C) Wild-type *SBH1 SBH2* S. cerevisiae and the indicated mutants were grown on YPD plates at the indicated temperatures for 3 days. (D) The *Δsbh1Δsbh2* S. cerevisiae strain was transformed with pRS415 without an insert (vector), wild-type *SBH1*, or the indicated phosphorylation site mutants and grown on SC without leucine containing 10 μg/mL calcofluor white at 30°C. A *Δkre6* mutant which is unable to grow on calcofluor white and its isogenic wild type were included as controls. Note that these are in a different strain background compared to the *SBH1* strain. (E) The indicated strains, including the untransformed *Δsbh1Δsbh2* S. cerevisiae strain (*Δsbh1/2*), were grown to early exponential phase and cell-wall proteins were extracted by high pH/DTT, resolved by SDS-PAGE (size range shown is roughly 10–150 kD), and visualized by Coomassie staining.

The biogenesis of both cell wall and capsule in C. neoformans relies on the secretory pathway, which begins with protein translocation across the ER membrane through the highly conserved Sec61 channel (11). An N-terminal hydrophobic signal peptide or a transmembrane domain targets proteins to this channel, which is formed by three proteins: Sec61, Sbh1, and Sss1 in yeast (Fig. 1A) and Sec61α, Sec61β, and Sec61γ in mammals (11, 12). *SEC61* and *SSS1* are essential in S. cerevisiae (13, 14). The 10 transmembrane helices of Sec61 form a protein-conducting channel in the ER membrane, stabilized by Sss1 which clamps around the Sec61 helix-bundle (Fig. 1A) (12, 15, 16). *SBH1* and its paralog *SBH2* are not essential, but deletion of both genes leads to temperature-sensitivity at 37°C in S. cerevisiae (17, 18). Sbh1/Sec61β is a tail-anchored protein associated peripherally with the Sec61 channel (19) (Fig. 1A). Its conserved transmembrane domain is sufficient to rescue the double mutant growth defect at 37°C, while the role of its divergent cytosolic domain containing multiple phosphorylation sites is poorly understood (18, 20, 21). As is common for proteins with intrinsically unstructured domains, Sbh1/Sec61β has multiple interactors: it binds to ribosomes and mediates association of the Sec61 channel with signal peptidase and signal recognition particle receptor (22–25). In mammalian cells, the Sbh1 homolog Sec61β also interacts with the Stimulator of Interferon Genes (STING); this interaction is required for STING's ability to stimulate interferon expression, thus linking the Sec61 channel to the innate immune response (26). Sec61 with Sss1 can translocate proteins into the ER on its own, but the cytosolic domain of Sec61β contacts secretory proteins prior to insertion in the cytosolic vestibule of the Sec61 channel and the presence of Sec61β stimulates import (23, 27, 28). Recently, null mutations in human SEC61B were identified as a cause for polycystic liver disease (29). The mutations led to a complete biogenesis defect specific for polycystin-1, a large complex transmembrane protein with a weak secretory signal sequence (29). Collectively, these data have led to the view that Sbh1/Sec61β mediates interaction of the Sec61 channel with other proteins in the ER membrane and that its cytosolic domain aids insertion of specific signal peptides or transmembrane domains into the Sec61 channel.

10.1128/mbio.03384-22.3 FIG S1 Alignments of full Sbh1 amino acid sequences. Protein sequences were extracted from Uniprot and aligned using Clustal Omega. (A) Sbh1 orthologues with N-terminal, proline-flanked S or T. Sbh1 = S. cerevisiae, proline-flanked N-terminal phosphorylation sites highlighted in yellow; Cryn, Cryptococcus neoformans, Sjap, *Schizosaccharomyces japonicus*, Hcap, Histoplasma capsulatum; Bder, Blastomyces dermatitidis; Cpos, *Coccidioides posadasii*; Afum, Aspergillus fumigatus; Anig, Aspergillus niger; Bfuc, *Botryotinia fuckeliana*; Sbra, *Sporothrix brasiliensis*. (B) Sbh1 orthologues without N-terminal, proline-flanked S or T. S. cerevisiae Sbh1 is shown for comparison; Sbh2, S. cerevisiae paralogue without N-terminal, proline-flanked S or T; Scry, *Schizosaccharomyces cryophilus*; Soct, *Schizosaccharomyces octoporus*; Cal, Candida albicans; Spom, Schizosaccharomyces pombe; Klac, Kluyveromyces lactis; Ylip, Yarrowia lipolytica; Ppas, Pichia pastoris; Hpol, *Hansenula polymorpha.* Download FIG S1, PDF file, 0.3 MB. Copyright © 2023 Santiago-Tirado et al. 2023 Santiago-Tirado et al. https://creativecommons.org/licenses/by/4.0/ This content is distributed under the terms of the Creative Commons Attribution 4.0 International license.

Phosphorylation is a frequent biological mechanism for regulation of protein activity or protein-protein interactions and a systematic analysis identified 63 kinases that play a role in the pathogenicity of C. neoformans (30, 31). One of these, Protein Kinase A (PKA), regulates the secretion of a subset of virulence factors (32). Regulation of protein secretion may be important during physiological transitions such as cell differentiation or development. Indeed Sec61β—the only subunit of the Sec61 channel that has been found to be phosphorylated thus far—is important for polarization of epithelial cells, and for development of Drosophila melanogaster embryos (33–36). Both mammalian Sec61β and yeast Sbh1 are phosphorylated at multiple sites in various combinations (21, 33; phosphosite databases). The phosphorylation sites all lie in the intrinsically unstructured cytosolic domain, and are not positionally conserved, with one exception: the proline-flanked site at T5 in S. cerevisiae is present in mammals and some birds, although not in lower vertebrates, invertebrates, or other nonpathogenic yeast species. This suggests that this phosphorylation site evolved at least twice by convergent evolution and thus likely has an important function (Fig. 1A; asterisk Fig. 1B) (21).

The 63 kinases required for virulence of C. neoformans include many proline-directed kinases (31, 37). When we aligned the N termini of Sbh1 proteins from nonpathogenic and pathogenic yeast, we noticed that—with the exception of Candida albicans—all pathogen Sbh1 proteins contain one or more potential proline-flanked phosphorylation site(s) in the N-terminal cytosolic domain, whereas Sbh1 proteins from nonpathogenic yeast species (Schizosaccharomyces pombe, Kluyveromyces lactis, Yarrowia lipolytica, Pichia pastoris, *Hansenula polymorpha*) and the S. cerevisiae homolog Sbh2 did not (Fig. 1B, pink; see Fig. S1 for full alignments). This led us to hypothesize that these phosphorylation sites in Sbh1 might be connected to virulence of the respective pathogenic species. In C. neoformans Sbh1, the proline-flanked phosphorylation sites are preceded by a potential PKA phosphorylation site (Fig. 1B, blue) and secretion of a subset of virulence factors in C. neoformans depends on PKA (32). We therefore decided to investigate the role of Sbh1 and its N-terminal phosphorylation sites in virulence of C. neoformans.

We found that, unlike in S. cerevisiae, mutations of the N-terminal Sbh1 phosphorylation sites (individually or in combination) do not affect C. neoformans growth in rich medium (YPD), and that even complete deletion of *SBH1* only inhibits growth when combined with high temperature and or cell-wall stressors. We also observed that in infection-like conditions the deletion mutant fails to properly regulate protein secretion. We have identified the Sbh1-dependent substrates and identified common characteristics of their ER-targeting sequences. Notably, the affected proteins include multiple virulence factors and the mutant is consequently avirulent in a mouse model of infection. Together, our results show that Sbh1 is critical for secretion of a subset of virulence factors and other proteins during C. neoformans infection, and is therefore essential for its pathogenicity.

## RESULTS

**Sbh1 is required for cell-wall integrity in S. cerevisiae and C. neoformans.** We first looked to the best characterized system, S. cerevisiae. In this organism, both genes that encode the Sec61 channel beta subunit (*SBH1* and *SBH2*) are required for growth on YPD at 37°C (Fig. 1C) (17, 18, 21). S. cerevisiae lacking *SBH1* and *SBH2* are also resistant to calcofluor white (Fig. 1D), which suggests either reduced chitin in the cell-wall or altered cell-wall architecture. Further, we observed increased extractability of proteins from the cell wall by alkaline buffer containing dithiotreitol, which also suggests structural defects in the wall (Fig. 1E). While mutation of the conserved Sbh1 phosphorylation site T5 to A on its own has no effect on S. cerevisiae growth or cell-wall integrity (21), mutation of both proline-flanked phophorylation sites to A phenocopied the absence of Sbh1 in S. cerevisiae. Mutation of both residues to phosphomimetic E had no effect (Fig. 1D and E).

**FIG 2:**
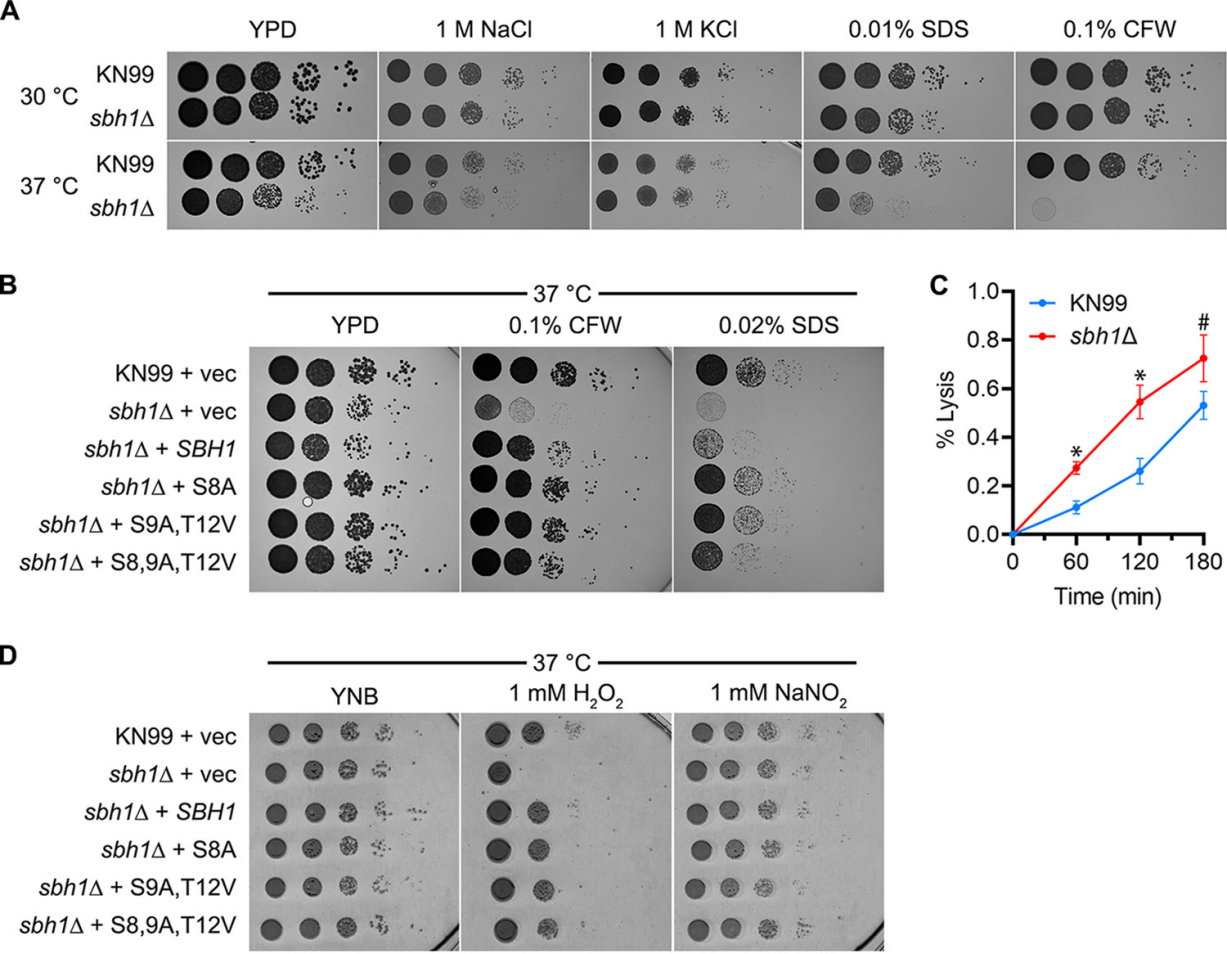
Sbh1 is required for cell wall integrity at high temperature in C. neoformans. (A) Serial 10-fold dilutions of wild-type (KN99) and *sbh1*Δ cells were grown at the temperatures shown on YPD alone or containing 1 mg/mL calcofluor white (CFW) or 0.02% SDS (SDS). (B) Wild-type (KN99) or *sbh1*Δ mutant cells were transformed with vector alone (vec) or plasmids encoding HA-tagged Sbh1 (SBH1), or Sbh1 with mutations in the indicated phosphorylation sites and then diluted as in panel A and grown at the indicated conditions. (C) Cells of the strains indicated were treated with lysing enzymes (see Materials and Methods) for the times shown and then assessed for % lysis with two-way ANOVA with mixed-effects analysis. * indicates *P* < 0.05; # indicates *P* = 0.25. (D) The indicated strains were grown as above in the presence of 1 mM H_2_O_2_ or 1 mM NaNO_2_ on YNB (pH 5) at 37°C. All experiments are representative of at least three biological replicate studies.

The phenotypes we observed in C. neoformans cells lacking their sole *SBH1* gene were quite distinct from those of S. cerevisiae. Specifically, they showed only subtle temperature sensitivity at 37°C on YPD (Fig. 2A, left panel) and no sensitivity to osmotic or cell-wall stressors at 30°C (Fig. 2A). At 37°C, however, the mutant cells were hypersensitive to SDS and barely grew on calcofluor white (Fig. 2A, lower panels; Fig. 2B, top two rows); they were also more sensitive than wild-type cells to cell-wall digestion by lysing enzymes (Fig. 2C) and showed increased sensitivity to oxidative stress (1 mM H_2_O_2_; Fig. 2D, top two rows), which is encountered in the setting of infection. Overall, we conclude that Sbh1 is required for cell-wall integrity in both the ascomycete S. cerevisiae and the basidiomycete C. neoformans, although specific manifestations of this requirement differ (see Discussion).

### Sbh1 N-terminal phosphorylation sites do not affect cell-wall integrity in C. neoformans.

As discussed above, we hypothesized that the N-terminal phosphorylation sites in cryptococcal Sbh1 were important for protein function. To test this, we first engineered a plasmid to express wild-type *SBH1* modified with a C-terminal HA tag. The tagged protein effectively complemented the sensitivity of *sbh1*Δ mutants to cell-wall stressors (CFW and SDS; Fig. 2B). We then modified this expression plasmid to encode Sbh1 that was altered to eliminate either the putative PKA site (Ser8 to Ala), two proline-flanked phosphorylation sites (Ser9 to Ala and Thr12 to Val), or all three sites. All of these polypeptides were expressed at similar levels, except for the version with a sole PKA site mutation, which was slightly less abundant (Fig. S2). We found that each of the mutated proteins fully complemented growth of *sbh1*Δ cells in the presence of CFW and also restored growth at 37°C in the presence of SDS (Fig. 2B). Interestingly, cells expressing mutant versions of Sbh1 grew slightly better on YPD and were more resistant to SDS than those expressing the WT protein (Fig. 2B). Each mutant protein also completely complemented the defective growth of the *sbh1*Δ mutant in the presence of oxidative stress (Fig. 2D). Together, these results suggest that, surprisingly, these phosphorylation sites are not required for Sbh1 function, as assessed by its role in cell-wall integrity.

10.1128/mbio.03384-22.4 FIG S2 Expression of wild type and phosphorylation mutants of Sbh1. An anti-HA immunoblot of wild-type or *sbh1*Δ cryptococcal cells expressing either vector alone (vec) or the indicated HA-tagged proteins is shown. 20 μg of lysate were resolved on a 4% to 20% gradient gel and transferred onto PDVF membrane, which was stained with Ponceau S. This blot is representative of three independent experiments. The numbers and lines on the left represent the protein ladder. The Sbh1-5XHA protein is ~16 kDa. Download FIG S2, PDF file, 1.1 MB. Copyright © 2023 Santiago-Tirado et al. 2023 Santiago-Tirado et al. https://creativecommons.org/licenses/by/4.0/ This content is distributed under the terms of the Creative Commons Attribution 4.0 International license.

### Sbh1-dependent secretory and transmembrane proteins are induced in conditions that mimic the host environment.

**FIG 3:**
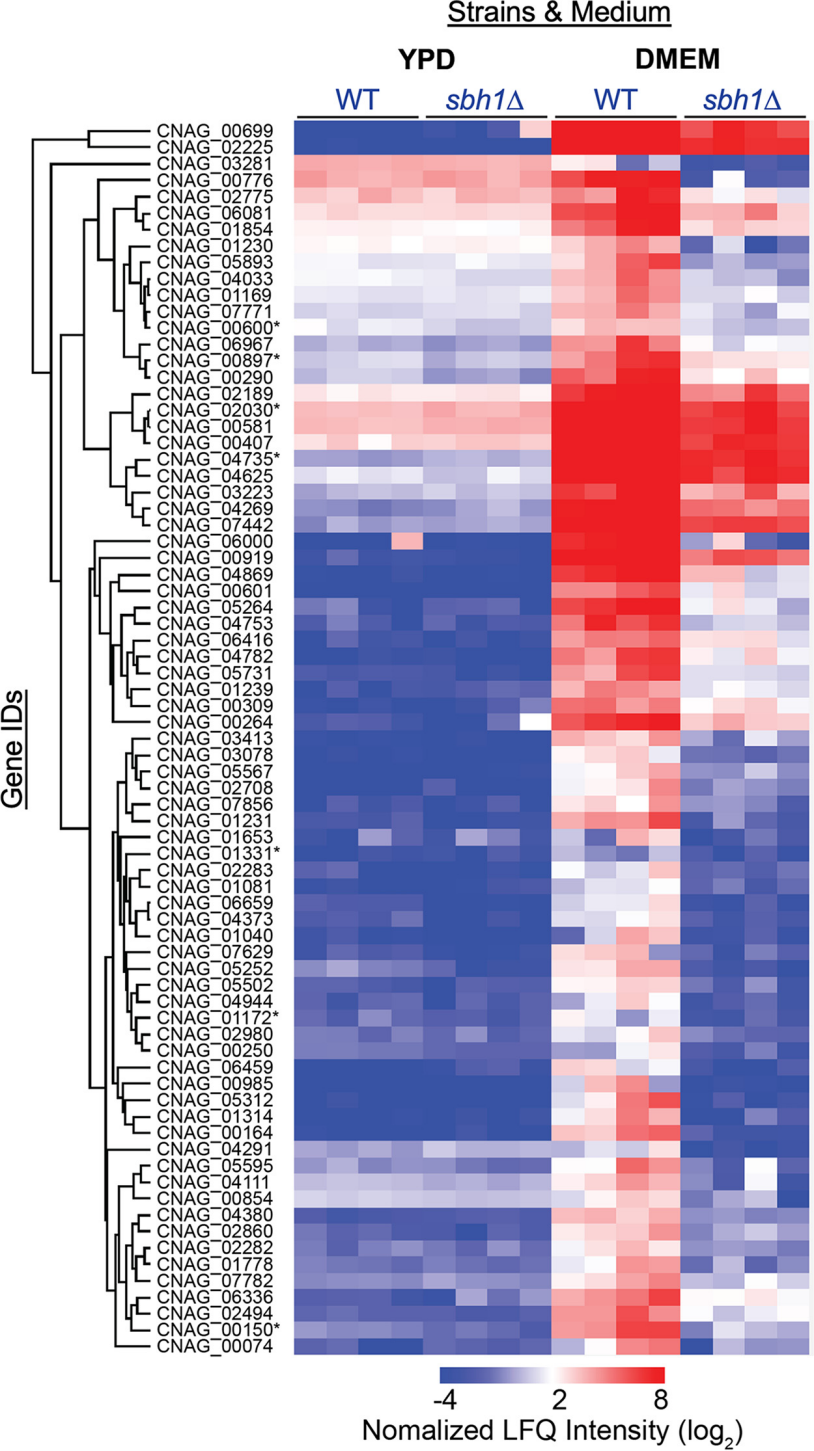
C. neoformans Sbh1 is required for the induction of secretory and transmembrane proteins in conditions that mimic the host. Proteomic analysis of wild-type and *sbh1Δ* C. neoformans grown in YPD at 30°C in room air or in DMEM at 37°C and 5% CO_2_. Samples were analyzed in quadruplicate. Gene IDs of known virulence factors are indicated by asterisks.

**FIG 4:**
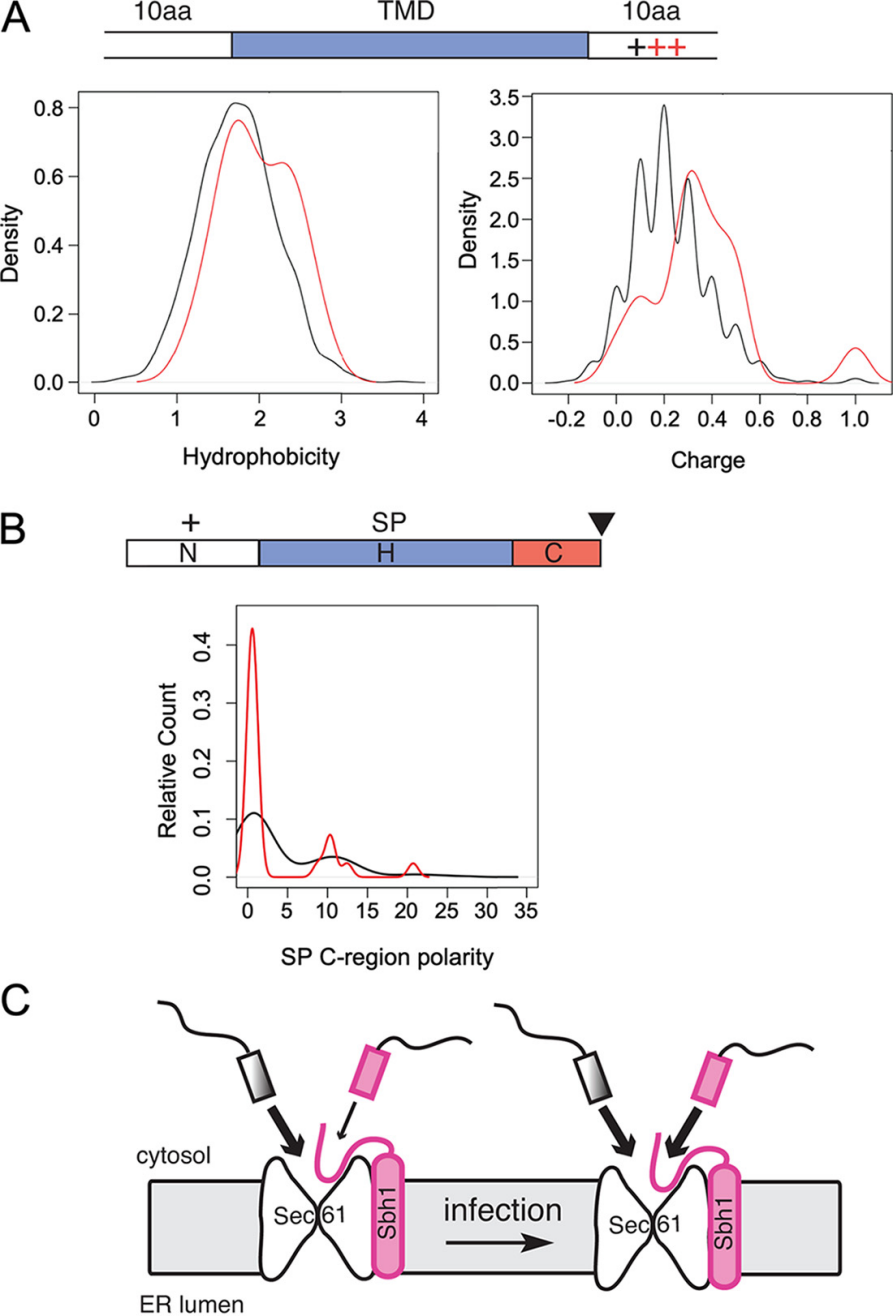
Secretory and transmembrane proteins that require Sbh1 for induction contain specific ER-targeting sequences. (A) Characterization of Sbh1-dependent transmembrane domains. Top, schematic of a typical transmembrane domain (TMD) with features specific for Sbh1-dependent TMDs highlighted in red. Hydrophobicity (left) and charges within 10 amino acids of TMDs (right) were analyzed for TMDs of Sbh1 dependently induced proteins (candidates, red) compared to all transmembrane proteins in the C. neoformans proteome (black). (B) Characterization of Sbh1-dependent signal peptides. The schematic on top shows a typical signal peptide (SP) with features specific for Sbh1-dependent SPs highlighted in red. Black triangle: signal peptidase cleavage site. SPs of Sbh1-dependently induced secretory proteins (red) were analyzed for N-region charge, H-region hydrophobicity and length, and C-region polarity and compared to all signal peptides in the C. neoformans proteome (black). The only difference found was in C-region polarity, shown in the graph. (C) Model for Sbh1 function in C. neoformans. Under rich medium conditions, proteins with Sbh1-dependent ER targeting sequences (magenta) play only a minor role for vegetative growth (left), and most proteins imported into the ER have Sbh1-independent targeting sequences (shaded gray). During infection (right), however, Sbh1-dependent ER import (magenta) becomes essential.

Sbh1 contacts signal peptides in the cytosolic vestibule of the Sec61 channel, so it can both guide secretory proteins into the channel and control their access to the secretory pathway. To address whether biogenesis of specific secretory or transmembrane proteins was compromised in the absence of C. neoformans Sbh1, we analyzed the proteomes of wild-type and *sbh1*Δ cells grown in either rich medium (YPD) at 30°C or in DMEM at 37°C and 5% CO_2_. The latter, termed 37D5 below, was chosen to mimic key features of the complex host environment. We detected no significant differences between wild-type KN99 and *sbh1*Δ strains in YPD at 30°C (Fig. 3), suggesting no critical role for Sbh1 in those conditions. In 37D5, however, we discovered a set of proteins that was specifically induced in wild-type cells, but not in the *sbh1*Δ mutant (Fig. 3; Table S1). This set included 32 proteins without secretory pathway targeting sequences, 21 transmembrane (TM) proteins, and 68 proteins with signal peptides (SP) or uncleaved signal anchors (SA) (Table S1). When we analyzed their ER-targeting sequences, we found that the transmembrane domains were slightly more hydrophobic than average for transmembrane proteins in C. neoformans; many also had a strong positively charged patch in close proximity to one side of the TM domain (Fig. 4A; Table S2). Some of the transmembrane domains of the Sbh1-dependently induced proteins also contained a high number of prolines and/or glycines in addition to the polybasic stretch adjacent to the transmembrane domain (Table S2). The Sbh1-dependent signal peptides were more heterogeneous, but most displayed reduced C-region polarity (Fig. 4B; Table S2). In addition, they were suboptimal in various ways which therefore defied bioinformatic analysis: some had limited hydrophobicity, some contained high numbers of proline and glycine residues which interfere with alpha-helix formation, and many had a charge bias problem (either no charge on either side of the hydrophobic core, a positive charge C-terminal instead of N-terminal of the hydrophobic core, or multiple positive charges at the N-terminus of the mature part of the protein) (Table S2). Because signal peptides insert as alpha-helices into the hydrophobic lateral gate of the Sec61 channel with the positively charged N-terminus toward the cytosol, any of these features will reduce translocation efficiency into the ER. We suggest that Sbh1 is required for the biogenesis of specific secretory and transmembrane proteins under host-like conditions. Our model is that because the targeting sequences of these proteins are suboptimal for Sec61 channel insertion, they rely on the enhanced insertion efficiency provided by the presence of Sbh1 (Fig. 4C) (23).

10.1128/mbio.03384-22.1 TABLE S1 Protein expression analysis in KN99 wild-type and *sbh1Δ* C. neoformans grown in YPD at 30°C or DMEM at 37°C and 5% CO_2_. Proteins are ordered by degree of Sbh1-dependence (log_2_ of wild type/*sbh1Δ* LFQ intensity). Where present, ER targeting sequences are indicated by amino acid numbers. SP, signal peptides; SA, uncleaved signal anchors; TM, first transmembrane domain. Ambiguous predictions due to limited hydrophobicity (TM) or suboptimal signal peptide parameters (SP) are indicated by '?'. Download Table S1, XLSX file, 0.02 MB. Copyright © 2023 Santiago-Tirado et al. 2023 Santiago-Tirado et al. https://creativecommons.org/licenses/by/4.0/ This content is distributed under the terms of the Creative Commons Attribution 4.0 International license.

10.1128/mbio.03384-22.2 TABLE S2 Signal peptides and transmembrane domains of proteins induced in DMEM in KN99 wild-type C. neoformans. Amino acid sequences of TM domains, signal peptides, and signal anchors are shown, including the flanking 10 amino acids on either side for TM or the first 10 amino acids of the mature domains for SP. Positively charged amino acid residues are highlighted in red. Download Table S2, DOCX file, 0.03 MB. Copyright © 2023 Santiago-Tirado et al. 2023 Santiago-Tirado et al. https://creativecommons.org/licenses/by/4.0/ This content is distributed under the terms of the Creative Commons Attribution 4.0 International license.

### Sbh1 is essential for virulence of C. neoformans.

**FIG 5:**
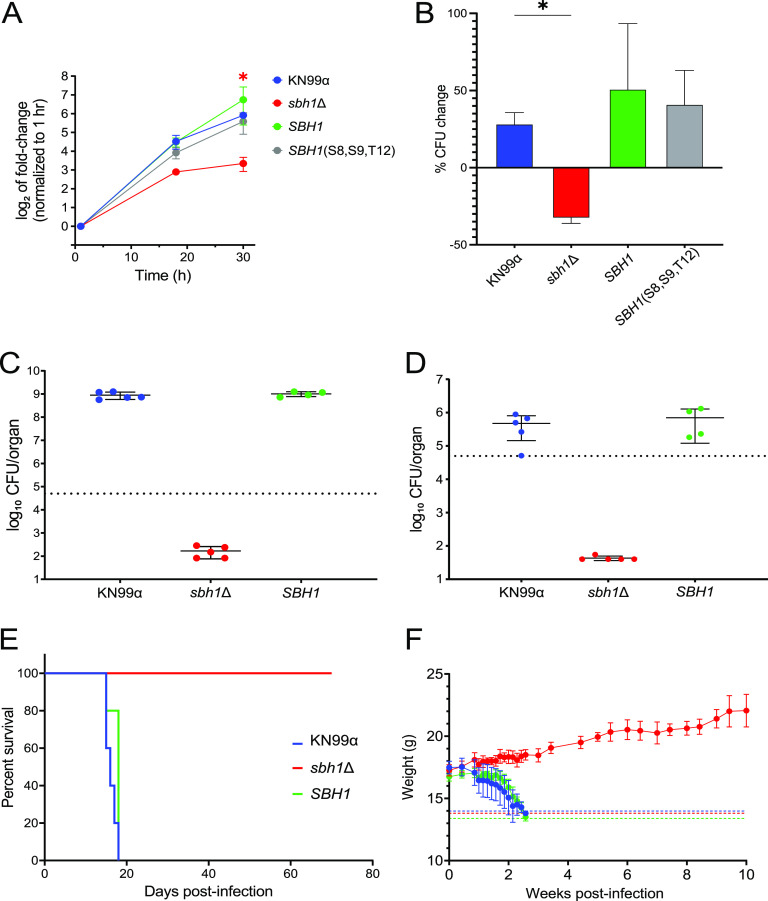
The C. neoformans *sbh1*Δ mutant is defective in growth in conditions that mimic the host or within host cells and is avirulent in mouse infection. (A) Fungi of the indicated strains were incubated in tissue culture medium (5% CO_2_, 37°C) for 1, 18, or 30 h and then plated on YPD for enumeration of CFU (CFU; shown normalized to the 1 h CFU value). Shown are mean and SEM values from 4 independent experiments. (B) Cells were incubated for 1 h with differentiated THP-1 cells (MOI = 1), during which time fungi were engulfed by the host cells. Samples were then vigorously washed to remove free cryptococci and host cells were lysed to measure CFU either immediately or after 30 h of incubation. % change was calculated as ([Final CFU − Initial CFU]/[Initial CFU] × 100). Shown are mean and SEM values from two independent experiments. For (A) and (B), * indicates *P* < 0.05 for *sbh1****Δ*** compared to KN99. (C and D) Total fungal burden at 14 days postinfection with the indicated strains, measured in lung (C) and brain (D). Each symbol represents CFU for one mouse; mean and standard deviation are also shown. Dotted line, original inoculum. (E) Mouse survival after intranasal infection with 5×10^4^ cells of the indicated strains. (F) Weight (mean and SD) of the mice from Panel E over time after infection. Any mouse that decreased to less than 80% of its initial weight (indicated by dashed lines) was humanely sacrificed and considered not to survive the infection.

The proteome analysis presented above shows that Sbh1 regulates the secretion of proteins that are induced by host-like conditions. This process is likely important for pathogenesis, as supported by our observation that *sbh1*Δ cells have a reduced ability to survive within host macrophages (Fig. 5A and B). To directly test the role of Sbh1 in virulence, we used a well-established mouse model for cryptococcal infection. At 14 days after intranasal infection, we observed a striking reduction in both lung and brain fungal burden in *sbh1*Δ-infected animals compared to wild type (Fig. 5C and D). Consistent with this finding, mice infected with wild-type cryptococci succumbed to infection within 18 days, while mutant-infected animals remained healthy and grew normally for at least 10 weeks (Fig. 5E and F). We conclude that *SBH1* is essential for C. neoformans virulence.

## DISCUSSION

In this work, we sought to identify factors regulating secretion of virulence-relevant proteins in the human pathogen C. neoformans. We specifically investigated the role of the nonessential ER protein translocation channel subunit Sbh1. Because the Sbh1 cytosolic domain contacts secretory signal sequences in the cytosolic vestibule of the channel prior to insertion into the channel, it can potentially aid or restrict protein import into the secretory pathway of specific proteins under specific growth conditions. This may be regulated by phosphorylation as Sbh1 in all species contains multiple phosphorylation sites which are only partially positionally conserved (Fig. 1A and B).

In S. cerevisiae, absence of *SBH1* and its paralog *SBH2* causes temperature sensitivity, although the ER translocation defects at the restrictive temperature are modest for most substrates investigated (Fig. 1C) (18, 38). We hypothesized that this phenotype could relate to defects in the cell wall for several reasons: other translocation mutants exhibit cell-wall defects (39); wall synthesis relies on secretory proteins; and alterations in this process often yield a temperature-sensitive phenotype (40). We investigated the effect of *sbh1* mutants on cell-wall integrity by assessing growth on calcofluor white (CFW; Fig. 1D) and extractability of cell-wall proteins by high pH/dithiotreitol treatment (Fig. 1E). CFW binds to chitin in the yeast cell wall and is toxic (41). *KRE6* encodes a glucosyl hydrolase required for beta-1,6-glucan synthesis; the deletion mutant is hypersensitive to CFW and is shown as a control. Cells lacking Sbh1 were slightly resistant to CFW (Fig. 1D), indicating a reduced amount of chitin in the cell wall, and displayed increased extraction of cell-wall proteins (Fig. 1E). S. cerevisiae cell-wall integrity also specifically required the two proline-flanked N-terminal phosphorylation sites at S3 and T5; mutation of these residues to nonphosphorylatable A phenocopied the cell-wall defects of the deletion mutant (Fig. 1D and E). The *sbh1 S3E/T5E* mutant behaved like wild-type (Fig. 1D and E). Proline-flanked phosphorylation sites in Sbh1 of other yeast species occur primarily in human pathogens (Fig. 1B), which suggested to us that phosphorylation at these sites may play a regulatory role in virulence. The absence of *SBH1* in C. neoformans resulted only in a very modest temperature sensitivity at 37°C (Fig. 1C versus Fig. 2A); although at high temperature, *sbh1Δ* C. neoformans also displayed a cell-wall defect (Fig. 2A to C). In contrast to S. cerevisiae, however, mutation of the N-terminal proline-flanked phosphorylation sites of C. neoformans Sbh1 individually or in combination did not phenocopy the cell-wall defect (Fig. 2B). As protein kinase A has been shown to be critical for C. neoformans virulence, and C. neoformans Sbh1 also contains a PKA site (Fig. 1B, blue), we also mutated this site alone or in combination with the proline-flanked sites, but again saw no effect on cell-wall integrity (Fig. 2B) (32). Surprisingly, the altered phosphorylation sites slightly enhanced cell growth, especially notable on SDS (Fig. 2B). Taken together, our data suggest that Sbh1 is important for efficient secretion of one or more enzymes or precursors for the cell wall in both S. cerevisiae and in C. neoformans, but that the role of the N-terminal proline-flanked phosphorylation sites in this process differs in the two organisms. Alternatively, the differential effect of the phosphorylation site mutations may reflect their distinct cell-wall compositions: whereas the S. cerevisiae cell wall is formed by β-1,3-glucan, β-1,6-glucan, chitin, and mannoproteins, in the C. neoformans cell wall, most chitin is deacylated to chitosan, and α-1,3-glucan is a major cell-wall component (10, 42).

We next asked which proteins were affected by absence of *SBH1* in C. neoformans. Using a proteomic approach, we identified a set of secretory and transmembrane proteins that were induced under 37D5 conditions in the wild-type strain, but not in the *sbh1Δ* mutant (Fig. 3; Tables S1 and S2). Many of the membrane proteins in this set were characterized by a strong polybasic patch adjacent to the first transmembrane domain (e.g., CNAG_1854, CNAG_5502, CNAG_6416), while the signal peptides of the secretory proteins were more heterogeneous, and had less polar C-regions than average (Table S2; Fig. 4A and B). This is reminiscent of the ER targeting signals identified by Ziska and colleagues in mammalian cells whose ER import was dependent on the Sec61 channel-interacting protein Sec63 and the ER-lumenal Hsp70 chaperone BiP (43). In S. cerevisiae the Sec63 complex composed of Sec63, Sec62, Sec71, and Sec72 promotes posttranslational protein import into the ER through the Sec61 channel by stabilizing the lateral gate in a partially open conformation (12, 44). It also plays a role in membrane protein topogenesis in both yeast and mammalian cells (45, 46). Sec63 is a polytopic membrane protein with an ER-lumenal J-domain that activates ATP-hydrolysis of the ER-lumenal chaperone BiP (Kar2 in yeast) (47). Kar2 contributes to protein import into the ER either by directly binding to the incoming protein or by promoting its folding (48, 49). As proteins dependent on Sbh1 in C. neoformans and proteins dependent on Sec63/BiP in mammalian cells have similar characteristics, our data suggest that Sbh1 and the Sec63 complex have overlapping functions in promoting ER import of specific proteins.

Signal peptides generally insert into the Sec61 channel with their N-terminus toward the cytosol and their N-terminal positive charge (Fig. 4B; “+”) contributes to this orientation (50). Many of the Sbh1-dependent signal peptides we identified in C. neoformans have no charge bias toward the N-terminus; in some, the charge bias is even reversed (Table S2). Sbh1 may help orient such signal peptides in the cytosolic vestibule of the Sec61 channel with the help of its cytosolic domain. The polar C-region of signal peptides contains the signal peptidase cleavage site (50). Most of the Sbh1-dependent signal peptides we identified have a very short C-region (Table S2), which may limit signal peptidase access unless the signal peptide or signal peptidase are positioned accurately by Sbh1. In order to efficiently insert into the Sec61 channel, the signal peptides of secretory proteins require an alpha-helical hydrophobic region (Fig. 4B and H, blue) (50). Many of the Sbh1-dependent signal peptides of C. neoformans contain multiple helix-breaking P or G residues within their hydrophobic helical core regions, and some have very short or very long hydrophobic H-regions (Table S2). These features would make it difficult for signal peptides to stably insert into the Sec61 channel lateral gate. Overexpression of an N-terminally truncated Sbh1 lacking most of its cytosolic domain complements the temperature-sensitivity and at least some of the translocation defect in *Δsbh1 Δsbh2 S. cerevisae* (18). Together with our data, this suggests that the Sbh1 transmembrane domain stabilizes a conformation of the Sec61 lateral gate that favors insertion of the helical hydrophobic core of signal peptides, even if they are suboptimal.

That Sbh1-dependent secretion is critical for virulence is illustrated by both the reduced intracellular fitness of *sbh1Δ* C. neoformans in macrophages and the effects in mice. In contrast to infection with KN99 wild-type C. neoformans, which resulted in a high burden in the lungs and death of all mice by day 18 postinfection, all mice infected with *sbh1Δ* C. neoformans survived for 70 days and even gained weight (Fig. 5), showing that the mutant strain was avirulent.

We hypothesize that the striking defect in virulence of the *sbh1* mutant reflects reduced secretion of multiple groups of proteins. For example, these could include proteins required for fungal growth in the host environment (e.g., metabolic genes or proteins involved in cell-wall synthesis), needed for virulence factor production, or directly involved in host damage. These categories may also interact; for example, mutations that alter cell-wall glycan synthesis also result in aberrant formation of the capsule (7, 8, 51, 52). To explore this idea, we reviewed the polypeptides whose secretion is most impaired in the absence of Sbh1 (Table S1), focusing on proteins with signal or transmembrane sequences that had been previously implicated in cryptococcal virulence. We identified eight such proteins, which had exhibited infectivity defects in a large-scale screen (53) and/or been individually studied and shown to contribute to virulence (Table 1). One of them, Cig1, has also been detected in the blood of mice infected with C. neoformans, leading to the suggestion that it could serve as a biomarker of infection (32). Notably, all of the corresponding genes are expressed in human CSF during cryptococcal meningitis, most at higher levels than in rich medium (Table 1 and [54]). Two proteins in this group (Pbx1 [55, 56] and Skn1 [57]) act in cell-wall synthesis, so their reduced secretion could contribute to the wall defects and sensitivity to host-induced stress that we observed in *sbh1* mutant cells. Another is required for capsule display (Cap60 [58]), although its biochemical function has not been characterized, and two others are proteases (CNAG_00150 and Mpr1 [59]). Many fungal proteases damage host tissues during infection (60) and Mpr1 further acts in cryptococcal crossing of the blood-brain barrier (59), which is required for cryptococcal dissemination to the brain and subsequent lethality. Supporting a role in infection for this group of proteins, six of the corresponding genes are expressed more highly in 37D5 than in rich medium (one other is modestly decreased); this pattern is particularly striking for *CIG1* and *MPR1*, whose expression levels increase over 100-fold (Table 1) (61). Notably, the evidence that these proteins are important for cryptococcal virulence derives from studies of individual gene deletion strains, which completely lack one of these target proteins. The situation in the *sbh1* mutant is different, in that many proteins are secreted less efficiently, but none (besides Sbh1) are absent. Nonetheless, the impaired secretion of multiple proteins with key roles in virulence, together with others in the functional groups above, provides abundant reason for the inability of *sbh1* to cause disease in a mammalian host.

**TABLE 1:** Sbh1-dependent proteins involved in cryptococcal virulence^*a*^

Gene ID	Gene name	Protein function	SP or TM	WT/*sbh1Δ* proteomics (log2-fold)	Virulence citations	24 h/0 h RNA-seq
CNAG_00150		Serine endoptidase, cell wall	SP1-23	5.24	53	1.68
CNAG_01653	*CIG1*	Cytokine-inducing glycoprotein	SP1-19	4.00	68	385.34
CNAG_01172	*PBX1*	Beta-helix containing protein	SP1-18	3.68	55, 53, 56	0.78
CNAG_04735	*MPR1*	Extracellular metalloproteinase (Mep1)	SP1-19	3.44	53, 59	122.79
CNAG_00897	*SKN1*	Glucosidase	TM134-58	3.04	57	3.43
CNAG_02030		Glyoxal oxidase	SP1-21	2.79	53	0.97
CNAG_00600	*CAP60*	Capsule-associated protein	SP1-33	2.65	58	1.97
CNAG_01331			SP1-18	2.62	53	13.45

a These proteins meet the following criteria: their expression in 37D5 was at least 2-fold higher in WT compared to *sbh1Δ* cells; they contain an SP or TM domain; and they are required for normal C. neoformans virulence in mouse infections. Note that this is likely a subset of such proteins, as most cryptococcal proteins have not been tested for roles in virulence; other candidates that meet the first two criteria are listed in Table S1. Proteomics results are shown as the ratio of expression in WT versus *sbh1*Δ cells in 37D5. RNA-seq results are shown as the ratio of gene expression at 24 h versus 0 h in 37D5 (61). Expression of a subset of these genes (CNAG_04735, CNAG_02030, CNAG_00897) was measured by qPCR; these studies confirmed that it is not affected by loss of *SBH1* (Fig. S3).

10.1128/mbio.03384-22.5 FIG S3 qRT-PCR analysis of selected genes in WT, *sbh1* mutant, and complemented strains grown in conditions that mimic the host (DMEM, 37°C, 5% CO_2_, 24 h). For each gene indicated, the plot shows mean and standard deviation of results from three biological replicates, each done in technical triplicate and normalized to *ACT1* expression. There were no significant differences in gene expression for any pairwise comparison between strains (all *P* > 0.2 by unpaired *t*-test). Download FIG S3, PDF file, 0.06 MB. Copyright © 2023 Santiago-Tirado et al. 2023 Santiago-Tirado et al. https://creativecommons.org/licenses/by/4.0/ This content is distributed under the terms of the Creative Commons Attribution 4.0 International license.

We conclude that C. neoformans Sbh1 is important for biogenesis of virulence factors whose specific ER-targeting sequences make these proteins dependent on Sbh1 for ER import (Fig. 4C). Recently, Sec61beta has also been shown to be critical for infection by the important fungal crop pathogen Magnaporthe oryzae, suggesting a more general role for Sbh1/Sec61beta in fungal infection (62). Because the intrinsically unstructured N-terminal part of the Sbh1 cytosolic domain is not conserved between yeast and mammals, our work may suggest a new specific target for developing drugs against this important human pathogen.

## MATERIALS AND METHODS

### S. cerevisiae methods.

S. cerevisiae strains are detailed in Text S1 and were grown on either yeast peptone dextrose (YPD) or synthetic complete medium without leucine, with or without 10 μg/mL calcofluor white (Sigma), at 30°C or 37°C, for 3 days. Cell-wall proteins were extracted from cells in early exponential phase in 100 mM Tris-HCl, pH 9.4, 10 mM DTT at 37°C, and TCA-precipitated for SDS-PAGE analysis as detailed in Text S1.

10.1128/mbio.03384-22.6 TEXT S1 Supplemental Methods. Download Text S1, PDF file, 0.1 MB. Copyright © 2023 Santiago-Tirado et al. 2023 Santiago-Tirado et al. https://creativecommons.org/licenses/by/4.0/ This content is distributed under the terms of the Creative Commons Attribution 4.0 International license.

### C. neoformans strains, growth conditions, and plasmids.

All strains were in the C. neoformans serotype A strain KN99α background, as detailed in Text S1, and were grown at 30°C on YPD with 100 μg/mL of nourseothricin or 100 μg/mL G418 as appropriate. After plasmids expressing the native *SBH1* gene or a version with 5 HA epitopes at the C-terminus were shown to complement phenotypic defects of the *sbh1* mutant, the tagged version was mutagenized to modify N-terminal motifs and electroporated into KN99α and the *sbh1*Δ strains as described in (63) and detailed in Text S1. Because of the heterogeneity in copy number associated with plasmids in C. neoformans, at least three independent colonies were picked and tested for each construct; all exhibited the same behaviors.

### Phenotypic assays and mass spectrometry.

Serial dilution plate assays and sensitivity to hypotonic lysis after exposure to lysing enzyme were assessed as detailed in Text S1 and (64). Total cellular proteome analysis was performed as in (65, 66) and Text S1. Results were compared to the C. neoformans reference proteome and deposited in the PRIDE partner repository for the ProteomeXchange Consortium with the data set identifier: PXD013894. A Student's *t* test was performed to identify proteins with significant differential expression (*P*-value < 0.05) (S_0_ = 1) between samples employing a 5% permutation-based FDR filter.

### Infection assays.

Fungal survival after engulfment by THP-1 (ATCC #TIB-202) cells *in vitro* was assessed as in (67), with minor modifications detailed in Text S1. For *in vivo* studies, 4- to 6-week-old female C57BL/6 mice were intranasally inoculated with 5 × 10^4^ cryptococcal cells and either followed by weight for survival studies or sacrificed at set times for organ burden measurement, as detailed in Text S1. All animal studies were reviewed and approved by the Animal Studies Committee of Washington University School of Medicine and conducted according to the National Institutes of Health guidelines for housing and care of laboratory animals.

### Data availability.

Raw data files are available in the PRIDE (Proteomics Identification Database); Project ID: PXD013894.
