# Supplementary material for: The ER Protein Translocation Channel Subunit Sbh1 Controls Virulence of Cryptococcus neoformans
Source: mBio. 2023 Feb 7;14(1):e03384-22. doi: 10.1128/mbio.03384-22 (PMC9973365; doi:10.1128/mbio.03384-22)
Supplement: FIG S1 [file mbio.03384-22-s0002.pdf]

A

```
CLUSTAL O(1.2.4) multiple sequence alignment


Sbh1            ------------------------------------------------------------  0
Cryn            ------------------------------------------------------------  0
Sjap            ------------------------------------------------------------  0
Cpos            ----------------------------------------MVSSSPLASGAESGNEAAKA  20
Afum            ---------------------------MHLLQ------ASARAASPLTSGAESGPDTKSS  27
Anig            ------------------------------------------------------------  0
Hcap            MPQGIYTTVDDERSLPGIREEFRSRNSSRLLQPILSFKTSPSNTMASGSGVESGAEGPKS  60
Bder            --------------------------------------------MASGSGPDSGAEGAKS  16
Bfuc            -------------------------------------MSSPRSASPVNSPV---------  14
Sbra            -------------------------------------MSSPRAASPTPAGA---------  14


Sbh1            ----------------MSSPTPPGGQRTLQKRKQGSS---Q--KVAAS-----APKKNTN  34
Cryn            ----------MADTKRPSSPSTPGQSSSFAVARPHGANAVRRRAPAAARPSSTRSAGAGG  50
Sjap            -----MSSPKSTPSVKSSPGAAPGGPKSQIRR---RAAAEK-SKEGKALPAGPRAAGANG  51
Cpos            SNPTGASTGSAASLNRPSSPTPPGGPRTALRR---RAAADHKESVRNARPASTRAAGAGG  77
Afum            --GAGAGASATGSVNRPSSPTPPGGPRAALRR---RAAADHKESLRNARPSSTRAAGAGG  82
Anig            --------SAVSSVNRPSSPTPPGGPRAALRR---RAAADHKESLRNARPSSTRAAGAGG  49
Hcap            ASGASS-GISGSALNRPSSPTPPGGPRTAMRR---RAAADHKESIRNARPASTRSAGAGG  116
Bder            STGVAS-GIAGSSLNRPSSPTPPGGPRTAMRR---RAAADHKESIRNARPASTRSAGAGG  72
Bfuc            -----A-----AVSGRPSSPTPPGGPKTAIRR---RAAADQKDKVANARPSSTRAAGAGG  61
Sbra            -----ASGASINRASSSSPPPPPGGARTQIRR---RAAADQKEKVANSRPTSTRAAGAGG  66
                                 *    **   :        :   :      :           .

Sbh1            SNNSILKIYSDE-ATGLRVDPLVVLFLAVGFIFSVVALHVISKVAGKLF-    82
Cryn            SSNTMLKLYTDSGEAGLKVDPFVVIVLSISFIASIFFLHITAKIIRAFTN    100
Sjap            STPTMLKLYTDE-TSGFKVDPVVVMVLSVGFIGSVFALHIIAKLMRKFSN    100
Cpos            SSGTMLKLYTDE-SPGLKVDPVVVLVLSLGFIFSVVGLHVIAKITRKFSS    126
Afum            SSGTMLKLYTDE-SPGLRVDPVVVLVLSLGFIFSVVGLHVIAKITRKFSS    131
Anig            SSGTMLKLYTDE-SPGLRVDPVVVLVLSLGFIFSVVGLHVIAKITRKFSA    98
Hcap            SSGTMLKLYTDE-SPGLKVDPVVVLVLSLGFIFSVVGLHVIAKISRKFA-    164
Bder            SSGTMLKLYTDE-SPGFKVDPVVVLVLSLGFIFSVVGLHVIAKITRKFSS    121
Bfuc            SSSTMLRLYTDE-SPGLKVDPVVVLVLSLVFIFSVVALHIIAKVTRKFSS    110
Sbra            SSSTMLRLYTDE-SPGLKVDPVVVLVLSLVFIFSVVALHIIAKITRRFSS    115
                *. ::*::*:*.   *::***.**:.*:: ** *:. **: :*:   :
```

B

```
Spom            MSSTKASGSVKNSAASAPGGPKSQIRRRAAVEKNTK-ESNSGPAGARAAGAPGSTPTLLK  59
Scry            MSSSKASGTVKNAGSSAPGGPKSQIRRRAAAEKTAK-DANTGPAGPRAAGAEGSTPTLLK  59
Soct            MSSSKASGTVKNAGSSAPGGPKSQIRRRAAAEKTAK-DSTTGPAGPRAAGADGSTPTLLK  59
Ylip            ----------MSTSAQVPGGPAAQMKRRNNAQRQEA-KASQRPTSTRSVGAGGSSSTMLK  49
Calb            ------MSASSSATNQAPGGLRSAVKRKTTQEKK-AQSSNATPLSTRSAGAGGSSSTMMK  53
Ppas            ------------MSTAIPGGQRTLAKRRAANLD----KKQDEPTSARSAGAGGSSSTMLK  44
Hpol            -------------MSSVPGGAKTLAKRKATQENKLKQQLQSQPTSTRAAGAGGSSSTMLK  47
Sbh1            -----------MSSPTPPGGQRTLQKRKQGSSQKVAA-SA-------PKKNTNSNNSILK  41
Sbh2            -----------MAASVPPGGQRILQKRRQAQSIKEKQ-AKQTPTSTRQAGYGGSSSSILK  48
Klac            ------------MDSSVPGGQRTLQKRRNAQLQKEKK-ANQTPASPRQAGFGGSSSSILK  47
                                 ***     :*:                        .*. :::*

Spom            LYTDEASGFKVDPVVVMVLSVGFIASVFLLHIVARILKKFASE   102
Scry            LYSDEATGFKVDPVVVMVLSVGFIASVFALHIVARILKNFASE   102
Soct            LYSDEATGFKVDPVVVMVLSVGFIASVFALHIVARVLKNFASE   102
Ylip            LYTDESQGLKVDPVVVMVLSLGFIFSVVALHILAKVSTKLLG-   91
Calb            LFTDEAQGLRVDPLVVLFLAVGFIFSVIILHVFAKITGKFTS-   95
Ppas            LYTDEAQGLKVDPLIVLVLAVGFIFSVIGLHVVAKLTGKLIN-   86
Hpol            IYTDESQGFKIDPLVVLVLAVSFIFSVVLLHVLAKLTGKFF--   88
Sbh1            IYSDEATGLRVDPLVVLFLAVGFIFSVVALHVISKVAGKLF--   82
Sbh2            LYTDEANGFRVDSLVVLFLSVGFIFSVIALHLLTKFTHII---   88
Klac            LYTDEANGLRVDPLVVLFLAVAFVFSVVALHVVAKVSGKIF--   88
                :::**: *:::* ::*:.*::.*: **. **:.::.   :
```

SUPPLEMENTARY FIGURE 1: Alignments of full Sbh1 amino acid sequences.

Protein sequences were extracted from Uniprot and aligned using Clustal Omega. (A) Sbh1 orthologues with N-terminal, proline-flanked S or T. Sbh1 = *S. cerevisiae*, proline-flanked N-terminal phosphorylation sites highlighted in yellow; Cryn *Cryptococcus neoformans*, Sjap *Schizosaccharomyces japonicus*, Hcap *Histoplasma capsulatum*, Bder *Blastomyces dermatitidis*, Cpos *Coccidioides posadasii*, Afum *Aspergillus fumigatus*, Anig *Aspergillus niger*, Bfuc *Botryotinia fuckeliana*, Sbra *Sporothrix brasiliensis*. (B) Sbh1 orthologues without N-terminal, proline-flanked S or T. *S. cerevisiae* Sbh1 is shown for comparison; Sbh2 = *S. cerevisiae* paralogue without N-terminal, proline-flanked S or T; Scry, *Schizosaccharomyces cryophilus*, Soct *Schizosaccharomyces octoporus*, Cal *Candida albicans*, Spom *Schizosaccharomyces pombe*, Klac *Kluyveromyces lactis*, Ylip *Yarrowia lipolytica*, Ppas *Pichia pastoris*, Hpol *Hansenula polymorpha.*
