# Supplementary material for: The ER Protein Translocation Channel Subunit Sbh1 Controls Virulence of Cryptococcus neoformans
Source: mBio. 2023 Feb 7;14(1):e03384-22. doi: 10.1128/mbio.03384-22 (PMC9973365; doi:10.1128/mbio.03384-22)
Supplement: FIG S2 [file mbio.03384-22-s0003.pdf]

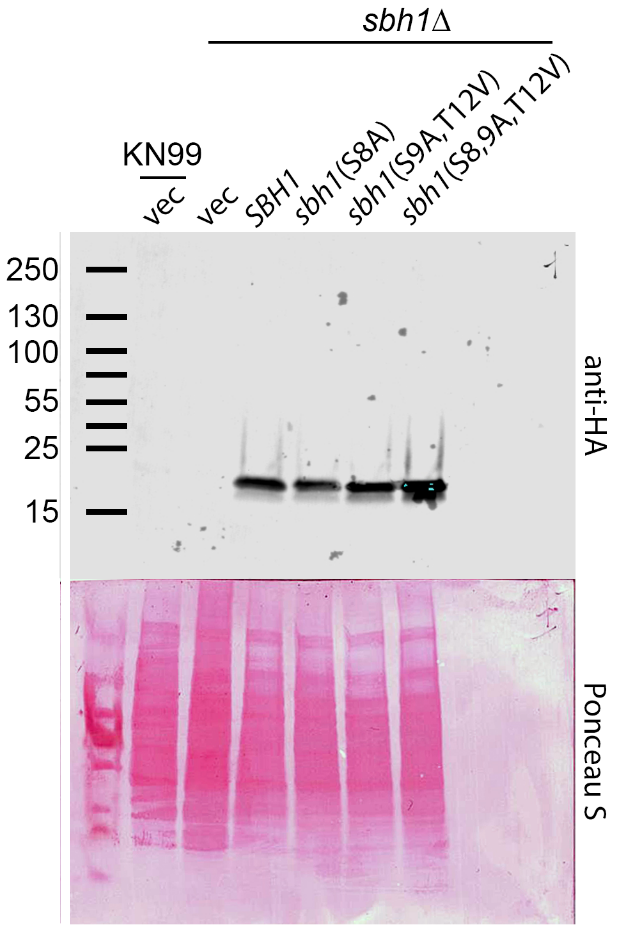

SUPPLEMENTARY FIGURE 2: Expression of wild type and phosphorylation mutants of Sbh1. An anti-HA immunoblot of wild-type or *sbh1*Δ cryptococcal cells expressing either vector alone (vec) or the indicated HA-tagged proteins is shown. 20 μg of lysate were resolved on a 4 – 20% gradient gel and transferred onto PDVF membrane, which was stained with Ponceau S. This blot is representative of three independent experiments. The numbers and lines on the left represent the protein ladder. The Sbh1-5XHA protein is ~16 kDa.
