# Supplementary material for: The ER Protein Translocation Channel Subunit Sbh1 Controls Virulence of Cryptococcus neoformans
Source: mBio. 2023 Feb 7;14(1):e03384-22. doi: 10.1128/mbio.03384-22 (PMC9973365; doi:10.1128/mbio.03384-22)
Supplement: TABLE S2 [file mbio.03384-22-s0006.docx]

**Signal peptides and transmembrane domains of proteins induced in DMEM in KN99 (wild-type) *C. neoformans***

Transmembrane Domains (TM)

CNAG_06336 AQQKARSRKR WFILLGGIILAVAAVVGIVVGVVV SQVNKNDDDN

CNAG_01854 GFQPKKKRNK WLWIGLPVLLICIILAAVLGGVL GSRASNDNKD

CNAG_04782 PPRWRYLRSF LPVIVLILLFFFLSSYSLLSH SKSPAAKQH

CNAG_00309 SHNNVRSGTS FAVRVTIATLIFLWTLLNINLVI SPFRQNSSISDL

CNAG_00074 RMNPRKRVNG IIPLILFIAAVIGFAVVSGIAIHSFV QVNGLGGGMG

CNAG_00985 IKRNRIQTTG VTMNLVLLAIFFFPLAFAQFGHF FQQFGPFGG

CNAG_00897 DITSARGWAN ALTLGILAGGGVMLFAGYPIISFYY GDSNSSGANTSG

CNAG_00854 SASSTRCLKK WSVRSLLVALLVSFYIWA NTINHKFYILDPPS

CNAG_06967 RERSATRLTS VLSRICIAFLIIVPTLAFAACYF GRTTLDKVRTW

CNAG_02494 KGGNTDWEAW YNAIFAFLVLGILVSIAGLYFYF RRKPVSYRSRISLK

CNAG_02283 HRYRRVVIKG LLLCLLILSAIVIRVSLGLW PHYLNSRSVRFEH

CNAG_05502 KKRGCYPSTRRGR LWFWGLIALTILAIVVIVAVCASVI PKNNSSSDAT

CNAG_06416 RTRPRSKSRK ILAILVAIIAFAGTIGVLAASGY SAVPSFSSKGG

CNAG_06000 RHSILPPQPT MLPLSLLPGLLALAPL ARAQVTATFPNAGAT

CNAG_06760 YRFSYTPTSP LLFLYSALTHNPHKIHY DQDWSVSKEE

CNAG_03078 RRGRGWITISG FSLPKPLLYLLAIPPLLILINLIAS TYNSARPHSHY

CNAG_03353 DRETILRPLR LTGLLGGYNIFAFAC GGGVSGQAGAVAL

CNAG_02592 SLVTKAIMSTTK IDLLIIGGGPAGL SAAITFSRLRRSC

CNAG_04753 GAKVLSWKSK LVYAGFTMGALATLAYTASHIL PSSSIAQASHL

CCP1 PSAPRSSNTG YIFAGLGVAAVGAAYYFY GTGRTEHDSTNKA

CNAG_02989 ARAEGQVGDLP FLRFMFHQFLLPFPFLTA APPTFWSAKVQPF

CNAG_00830 KFKGNNSSSSVEG LLFMAFAAVQFLFWRVFM RLDYQCPPHLTFT

CNAG_03063 SLSTNAGLPS LQIIIPASSTVTLFIAFQ PSVSLPPFPLSPL

CNAG_04085 RTVDWKTAKIG IIGLGSIGSHLAFLLSSIGAT SILYHSRRPSLHAA

CNAG_02702 RLIRRTLEEGE AYVVISLVGVIIGVSAALISII TTWLSDIKLG

CNAG_05193 VIRERTTSLYQ FLIRLAGVVGGVWTVAAFALRV FNRAQREVSK

CNAG_01637 TMEDVKIKTRT GALLTFISLSIILTSVMLEFI DYRRIHLEPS

**Signal peptides and transmembrane domains of proteins induced in DMEM in KN99 (wild-type) *C. neoformans***

Signal Anchors (SA)

CNAG_05293 MSLPNA FVTLLTTSSYLPGALVLLHALH DLHPAPRDFQ

CNAG_02980 HLPSNRRRTS ILLSLLALLLLLAAGLSLALVV KHRHNEPTDFLE

CNAG_05567 MTARQT IIIVSVVSLLLLYLFVHHI TSSLEPSLPNAP

CNAG_05726 DLYLDPSIRD WVLIPITLIMLLVGVLRHYV TQFLNSAPKKQ

CNAG_01307 MTLYYS ICFALLMSELSLFCTIVCPMPFAI RKKMFHFLSE

Signal Peptides (SP)

CNAG_04735 MRSSALIALLPFLATLTAA RPHHREDKHS

CNAG_02225 MRLLIPFFIVPLAIA RALPAFPTVD

CNAG_04869 MRWLNIFPLLLPFVADA SPLQPRRQAT

CNAG_05264 MPVLSNLISFLPFLAIAHA ATSDEWRSRS

CNAG_00699 MLVNNLFYLIAALVTSAFVVA QDDGWHIDYV

CNAG_02189 MLVHQAALALIPLFALLPVYA LDADAMRSRS

CNAG_02030 MTSLNMATLLALLPLLSLVVA SPSPTPQDGT

CNAG_03146 MLCCFCHRSLSVFNCLFVLRSPTRA SLGKIEIPLL

CNAG_00581 MKTSAILIAALSAAASVEA GIHRMKLEKQ

CNAG_07771 MRARLLALLGLSGSVWA TPALFTVEDM

CNAG_00407 MYSAILLSLLPLLAAA HRGPSRNLPN

CNAG_04269 MLITPAILALPLLASA VPTAREQLAF

CNAG_00290 MPSLLLPLILHLLSSLTNTAAA AAVPPSSPAL

CNAG_00919 MCSKVVSAVLLAFALGSVI EAAREPHGLR

CNAG_00264 MKLLPLALVVASTLPSALS WGAAGHEMVA

CNAG_02775 MFALKSILVTSLITSTALA HFTLDYPQRS

CNAG_03486 MNISHPIPTTMASRSFLSLLVALFVAICFVLSPGADA AKGPVITNKV

CNAG_00264 MKLLPLALVVASTLPSALS WGAAGHEMVA

CNAG_02189 MLVHQAALALIPLFALLPVYA LDADAMRSRS

CNAG_07442 MKLVPLLIPFIATTVTASLA GEALSWASQL

CNAG_04625 MRFTAASLLLLPLAALA SPIAQPSLAP

CNAG_00150 MVAFHASSALLSFALLATGFANA FLSDDIKRGSDS

CNAG_00250 MLAANLISLVLVLPTLALA RHNSPFNGHSPS

**Signal peptides and transmembrane domains of proteins induced in DMEM in KN99 (wild-type) *C. neoformans***

CNAG_00776 MISKVAVGAAAALMAGVANVNA QVTATGTMGPTN

CNAG_00588 MLFFATLLPLLALVSA APFTKRYTGAKI

CNAG_00164 MTMLLGAFISSMICLLSHIPTAFA FGLQNYPNLFF

CNAG_00601 MLFPALALLCPVLVA AHGQLSWVQVGTGP

CNAG_04033 MIRSSLLWLTIIATLALLSLTLA VKSEDFKQCSQ

CNAG_02860 MLVSNVVFPLFTLSFAVPAVSA MRLNPAHAKR

CNAG_02708 MPPPSLTSSSTRFRVGFFVLLLAVSLSLT HFTPSFTSHRS

CNAG_01314 MRFPLLLPFAALELPIPTAP LEWKQANFLS

CNAG_01040 MWSKILTTALLVFALGSVVGA ARDPLGLRGRR

CNAG_01233 MLPTFPRPISISSLLISFIHVSSI TVVASTAAPA

CNAG_01172 MLFPLSLLLHLTSTVAA ATTKCLVSGDQD

CNAG_00600 MPPQSFPLTVAPQTQRLAIRAALAILGLFILRA LFSSSKSPEEIQSH

CNAG_07629 MRSRLLASLFTLALSVASSEA ISSTGQMPLNG

CNAG_05567 MTARQTIIIVSVVSLLLLYLFVHHITSS LEPSLPNAPSRD

CNAG_01230 MIPSTAAALLTLTAGAAFA HTGCGGHEIGR

CNAG_01169 MAKASVVLFLSATTRSLA RYLMKASIPFPP

CNAG_05252 MLPTTLFFLLTLALLGGCLA ESLYSVLGVRK

CNAG_05893 MKYALFALALAGAVSA QSNSTANSTESILI

CNAG_05312 MSSLHHLVPLLFLIAPALS QYTATYSPSSLP

CNAG_03413 MSPFLTAALLSTLLSFVPSPVSA VTLESIIDAYGLS

CNAG_01331 MLFGTLAYLALTITYGYS LTVPQAHRETLE

CNAG_03223 MHSTAIIASFLASLALSNA IKVESPNKDTV

CNAG_04111 MRRNPTLSLLSLTALTAISGVNA AEPEDSLTSVI

CNAG_04944 MRSPGPSLLLLAALPLASA THLSNKLQDKV

CNAG_04291 MLLPVITPLLLAAASYA QQSTPRVLVYSAT

CNAG_07782 MTKSLYAFFSIILLFSVSSSVAA SPQAQHHQHAMASS

CNAG_01231 MYGSLGVSILLMAATPIVLA KIQEGGGYSQ

CNAG_07856 MLLPLIISTAIGVAPFARA YLVNSSDYSSG

CNAG_06659 MLFNGLLEAVSLSLPFFASPSPLSA KPDINVVPLPRH

CNAG_05731 MLSPLTLLHALSLAALAALPAQA QLANTFQYVGLSGVSA

CNAG_01653 MIFNRFTFTAAMAASAASA ISVQRRAQITD

CNAG_04380 MVNAHILTVLSILPLSLA TLPEPDPHNGLSL

**Signal peptides and transmembrane domains of proteins induced in DMEM in KN99 (wild-type) *C. neoformans***

CNAG_02282 MRTSPHTCVLLLCAASAIA TPQHQAPIQLP

CNAG_06459 MRSLSVFAIAICARSAVA GQSPFTFGRAPQ

CNAG_04373 MLTSIIAALPLLASLPLSSA YAVSDDAALES

CNAG_05595 MFAVAALASLLSAIAVKA VPCVQFDSSWNL

CNAG_01778 MFDFIITFFIAAAILAAVMALSVA ITLPFAGALVRW

CNAG_06081 MLAPLALLPLLSLALPQVSA DTRPGGLPHN

CNAG_01239 MYGHLSLSALSLFAVVAA APFRESWLQPRD

CNAG_02592 MPSLVTKAIMSTTKIDLLIIGGGPAGLSAA ITFSRLRRSC

CNAG_01081 MKVIVLGASGFIGHRVALA FVQAGHFVWG

CNAG_01601 MYIPGPLRLSSYFLPFISSPSPPAQS SPDTRTISFKP
