# Supplementary material for: The ER Protein Translocation Channel Subunit Sbh1 Controls Virulence of Cryptococcus neoformans
Source: mBio. 2023 Feb 7;14(1):e03384-22. doi: 10.1128/mbio.03384-22 (PMC9973365; doi:10.1128/mbio.03384-22)
Supplement: FIG S3 [file mbio.03384-22-s0004.pdf]

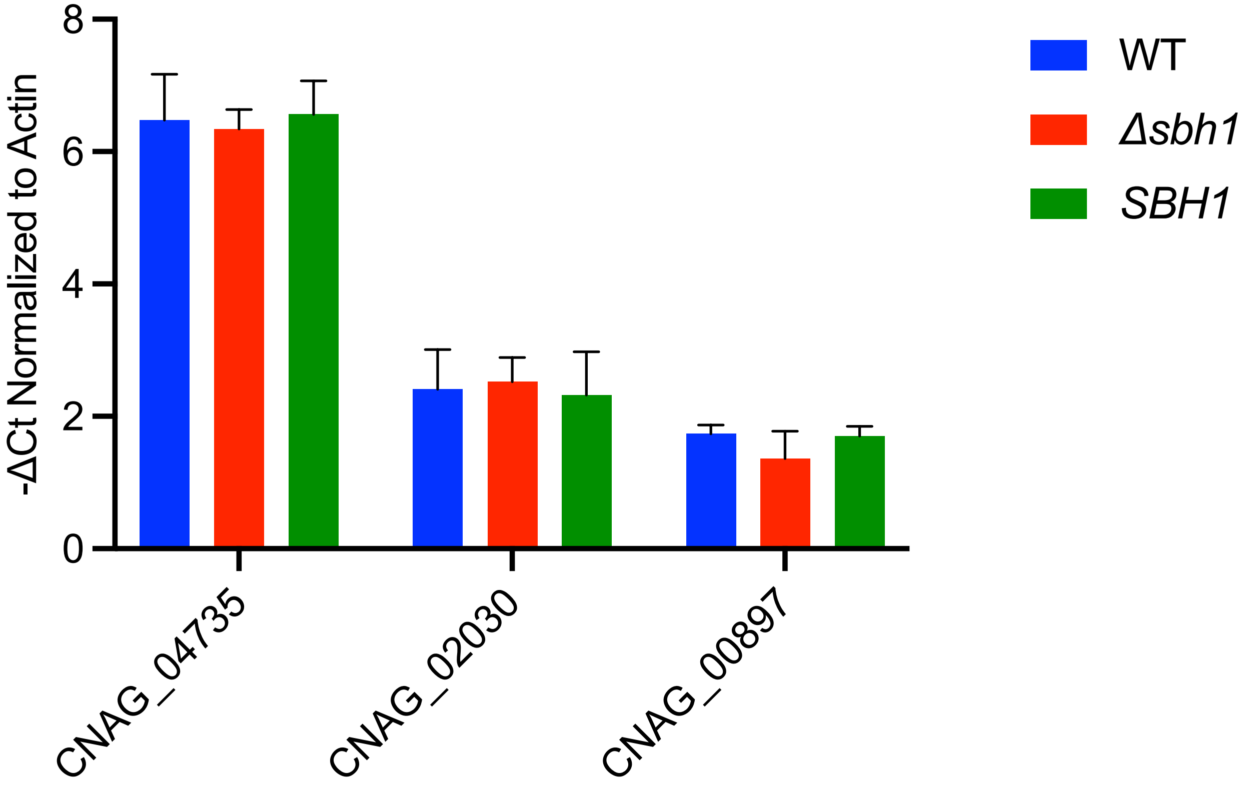

SUPPLEMENTARY FIGURE 3: qRT-PCR analysis of selected genes in WT, *sbh1* mutant, and complemented strains grown in host-like conditions (DMEM, 37°C, 5% CO_2, 24 h). For each gene indicated, the plot shows mean and standard deviation of results from three biological replicates, each done in technical triplicate and normalized to *ACT1* expression. There were no significant differences in gene expression for any pairwise comparison between strains (all $P > 0.2$ by unpaired T-test).
