# Supplementary material for: The ER Protein Translocation Channel Subunit Sbh1 Controls Virulence of Cryptococcus neoformans
Source: mBio. 2023 Feb 7;14(1):e03384-22. doi: 10.1128/mbio.03384-22 (PMC9973365; doi:10.1128/mbio.03384-22)
Supplement: TEXT S1 [file mbio.03384-22-s0001.pdf]

Santiago-Tirado *et al* **SUPPLEMENTAL METHODS**

<u>*S. cerevisiae* strains and growth conditions</u>

NY179 (*SBH1 SBH2 MAT* a *leu2-3,112 ura3-52*), H3223 (MAT a *KanMx::sbh1 leu2-3,112 ura3-52 GAL*$^+$), H3203 (MAT a *HphMx::sbh2 leu2-3,112 ura3-52 GAL*$^+$), and H3231 (MAT a *KanMx::sbh1 HphMx::sbh2 leu2-3,112 ura3-52 GAL*$^+$) were gifts from Jussi Jäntti (Helsinki University, Finland) and were used to characterize Sbh1p phosphorylation sites (Toikkanen et al., 1996; Feng et al., 2007). *SBH1* cloned into pCR2.1-TOPO plasmid was kindly provided by Jussi Jäntti. The QuickChange Site-Directed Mutagenesis Kit (Stratagene, UK) was used to introduce single or multiple base mutations in *SBH1.* Substitutions were verified by sequencing. Mutated *sbh1* was excised from pCR2.1-TOPO plasmids using EcoRV/BamHI, and subcloned into pRS415. For growth assays cells were grown on YPD or synthetic complete medium without leucine and with or without 10 µg/ml calcofluor white (Sigma) at 30°C or 37°C for 3 days.

<u>*S. cerevisiae* cell wall extraction</u>

Cells were grown in synthetic complete medium without leucine to early exponential phase and 2 x 10^8 cells were harvested by centrifugation (5 min, 10,000 g, room temperature), resuspended in 100 µl 100 mM Tris-HCl, pH 9.4, 10 mM DTT, and incubated for 15 min at 37°C shaking at 800 rpm in a Thermomixer (Eppendorf). Samples were chilled for 1 min on ice, followed by sedimentation of the cells in a refrigerated Eppendorf centrifuge at maximal speed for 1 min. 90 µl of each supernatant were transferred to a new tube, carefully avoiding the pellet, and centrifuged again as above. 80 µl of each supernatant were transferred to a fresh tube and proteins precipitated by adding an equal volume of ice-cold 20 % trichloroacetic acid. Samples were mixed thoroughly and incubated on ice for 30 min. Proteins were sedimented by centrifugation at 4°C for 15 min and the pellets washed with ice-cold acetone, dried, and resuspended in 20 µl 2x SDS sample buffer by heating for 10 min to 95°C with agitation. Proteins were resolved on 4-14% Bis-Tris gels (NuPage) run in MOPS buffer and revealed by Coomassie staining.

<u>*C. neoformans* strains and growth conditions</u>

All strains used were in the *C. neoformans* serotype A strain KN99α background. The *sbh1*Δ deletion in this background used here was obtained from a library of partial gene deletions (generated by the Madhani group (Liu et al, 2008)) and available from the Fungal Genetics Stock Center at Kansas State University, Manhattan, KS) and confirmed by PCR. Fungal stocks were maintained at -80°C and grown at 30°C on yeast peptone dextrose (YPD) media with antibiotics as appropriate (100 µg/mL of nourseothricin (clonNAT, WERNER BioAgents, Germany) or 100 µg/mL G418 (Geneticin, Life Technologies, USA)).

<u>*SBH1* plasmid construction</u>

For plasmid construction, the *SBH1* gene with its promoter and terminator sequences was amplified and cloned into ApaI/SpeI-digested pIBB103 (Skowyra and Doering, 2012). In parallel, a similar fragment lacking the terminator sequences and stop codon was amplified and cloned into ApaI/SpeI-digested pFS5XHA (pIBB103 containing 5 HA epitopes followed by the *TRP1* terminator; this study). After both versions were confirmed to complement phenotypic defects, the tagged version was used for all subsequent analyses. This construct was used as template for mutagenesis of the N-terminal motifs of *SBH1* by using overlapping primers containing the codon changes and the forward and reverse primers used above. The resulting product was digested, cloned into pFS5XHA, and the whole insert sequenced to confirm mutations. These plasmids, together with an empty plasmid control, were electroporated into KN99α and the *sbh1*Δ strains as detailed below.

<u>Electroporation of *C. neoformans*</u>

Plasmid transformation was done by electroporation as described in Skowyra and Doering (2012). In brief, *Cryptococcus* cells patched on YPD agar plates with appropriate antibiotics were transferred into 5 ml of YPD liquid medium with antibiotic and cells were grown overnight at 30°C with shaking at 250 rpm. The overnight culture was diluted to an OD_{600} of 0.05 in 50 ml of fresh YPD medium (note that this OD_{600} value was measured in a multiplate reader and was not corrected for pathlength). After two doubling times the cells were transferred into conical tubes and sedimented, washed twice with cold dH_2O, and resuspended thoroughly in 50 ml cold EB buffer (10 mM Tris-HCl, pH 7.5, 1 mM $MgCl_2$, and 270 mM Sucrose) with 4 mM DTT. After incubation on ice for 15 min, the cells were pelleted, washed with 50 ml EB buffer without DTT, and resuspended in 1 ml EB buffer for counting. Cells were adjusted to 300 million cells per 100 µl, mixed with at least 1 µg of DNA (in no more than 1/10 of the total volume) and the mixture transferred into ice-cold 2 mm gap cuvettes. A Biorad electroporator was used with the following settings: V = 500v, R = infinity ohms, C = 25µF; the time constant should be 15 – 25 ms. Immediately after electroporation, 900 µl YPD without antibiotics was added and the cell suspension was transferred to a culture tube and incubated at 30°C for 3-4 h for recovery before various dilutions were spread on YPD plates with antibiotics to select transformants. Because of the heterogeneity in copy number associated with plasmids in *C. neoformans*, at least three independent colonies were picked and tested for each construct. All exhibited the same behaviors.

<u>Phenotypic analysis</u>

For stress plating, strains to be tested were grown overnight in YPD with antibiotic as appropriate, diluted to an OD_{600} of 0.05 in fresh YPD, and grown for two doublings. The cultures were then adjusted to 2 x 10^9 cells/ml, serially diluted (10-fold) and 5-µl aliquots were spotted onto the phenotyping plates described in the text. These plates did not include antibiotics because in combination with stressors they inhibit cell growth. Plates were incubated at 30°C and 37°C for 3-4 days.

The assay for sensitivity to lysing enzymes was adapted from Gerik et al. (2005). Briefly, overnight cultures of wild type and mutant strains in YPD medium were washed once with PBS and once with citrate buffer (10 mM, pH 6.0). Cells were resuspended in citrate buffer and the OD_{600} was normalized to 1.0/ml with the same buffer. 1 ml aliquots were then subjected to centrifugation and the pellets (one per time point) were resuspended in a solution of lysing enzyme (final concentration 25 mg/ml; Sigma, L-1412) and incubated at 37°C with gentle agitation. At each desired timepoint one aliquot per strain was sedimented and resuspended in the same volume of distilled water (in the absence of lysis the OD_{600} should remain ~1.0). The assay was performed independently three times.

Mass spectrometry analysis

For total cellular proteome analysis, cells were collected by centrifugation at 3,500 rpm for 10 min and the pellets were washed twice with cold PBS before being subjected to acetone precipitation and enzymatic digestion (Geddes et al., 2016; Ball and Geddes-McAlister, 2019). For secretome analysis, supernatant fractions from *C. neoformans* WT and *sbh1*Δ cell cultures were collected and filtered using 0.22 µM syringe filters and subjected to in-solution trypsin digestion (Ball and Geddes-McAlister, 2019). Digestion was stopped by the addition of 10% v/v trifluoroacetic acid (TFA) and the acidified peptides were desalted and purified according to the standard protocol (Rappsilber et al., 2007). Approximately 50 µg of sample was loaded onto StageTips and stored at 4°C until LC-MS/MS measurement.

For LC-MS/MS measurement, samples were analyzed by nanoflow liquid chromatography on an EASY-nLC 1200 system (ThermoFisher Scientific) on-line coupled to a Q Exactive HF-X quadrupole orbitrap mass spectrometer (ThermoFisher Scientific). An in-line 75 µm x 50 cm PepMap RSLC EASY-Spray column filled with 2 µm C18 beads (ThermoFisher Scientific) separated peptides using linear gradients from 3% to 20% Buffer B (80% acetonitrile) over 18 min and from 20% to 35% Buffer B over 31 minutes, followed by a steep 2 min ramp to 100% Buffer B for 9 min in 0.1% Formic acid at a constant flow of 250 nl/min.

Raw files were analyzed together using MaxQuant software (version 1.6.0.26) (Cox & Mann, 2008). The derived peak list was searched with the built-in Andromeda search engine (Cox et al., 2011) against the reference *C. neoformans* H99 proteome downloaded from Uniprot (http://www.uniprot.org/) (Aug. 18, 2018; 7,430 sequences). The following parameters were included: trypsin enzyme specificity with a maximum of two missed cleavages, a minimum peptide length of seven amino acids, fixed modifications, including carbamidomethylation of cysteine, and variable modifications, including, methionine oxidation and N-acetylation of proteins and split by taxonomic ID. Peptide spectral matches were filtered using a target-decoy approach at a false-discovery (FDR) of 1% with a minimum of two peptides required for protein identification. Relative label-free quantification (LFQ) and match between runs were enabled;

the MaxLFQ algorithm used a minimum ratio count of 1 (Cox et al., 2014). The mass spectrometry proteomics data have been deposited in the PRIDE partner repository for the ProteomeXchange Consortium with the data set identifier: PXD013894.

Statistical analysis of the MaxQuant-processed data was performed using the Perseus software environment (version 1.6.2.2) (Tyanova et al., 2016). Data were prepared by filtering for reverse database matches, contaminants, and proteins only identified by site, followed by $\log_2$ transformation of LFQ intensities. Filtering for valid values (three of four replicates in at least one group) was performed, and missing values were imputed from the normal distribution (width, 0.3; downshift, 1.8 standard deviations). A Student's *t*-test was performed to identify proteins with a significant differential expression (*p-value* < 0.05) ($S_0 = 1$) between samples employing a 5% permutation-based FDR filter.

In vitro macrophage survival assay

Fungal survival after engulfment by THP-1 (ATCC #TIB-202) cells *in vitro* was assessed as in Santiago-Tirado et al. (2015) with only minor modifications: First, since the cryptococcal strains were grown in G418 for plasmid maintenance, they were washed extensively with PBS to remove all traces of drug before being opsonized, adjusted to an MOI of 10, and added to the THP-1 cells. Second, for CFU determination, 0.1% Triton-X100 (which had no effect on the cryptococcal cells) was used instead of SDS to lyse the THP-1 cells.

Infection studies

All animal studies were reviewed and approved by the Animal Studies Committee of Washington University School of Medicine and conducted according to the National Institutes of Health guidelines for housing and care of laboratory animals. Strains to be tested were cultured overnight in YPD medium, collected by centrifugation, washed in PBS, and diluted to 10^6 cells/ml in PBS for intranasal inoculation (50 µl) into 4-6 week-old female C57BL/6 mice (National Cancer Institute) that had been anesthetized with a combination of ketaset-HCl and xylazine. Initial inocula were plated to confirm CFUs. To assess long-term survival, infected animals were weighed 1 h post-infection and at least every other day afterwards. Mice were sacrificed if their weight fell below 80% of peak (an outcome which in this protocol precedes any signs of disease) or at the end of the study (10 weeks). To measure organ burden, infected mice were monitored as above for 14 days, at which point lungs and brains were harvested from all mice, homogenized in PBS, and serial dilutions of the homogenate plated on YPD agar for enumeration of CFU.

REFERENCES

Ball B, Geddes-McAlister J (2019). Quantitative Proteomic Profiling of *Cryptococcus neoformans*. *Curr Protocols Microbiology* 55:e94.

Cox J, Hein MY, Luber CA, Paron I, Nagarj N, Mann M (2014). Accurate proteome-wide label-free quantitation by delayed normalization and maximal peptide ratio extraction, termed MaxLFQ. Mol Cell Proteomics 13:2513-26.

Cox J, Mann M (2008). MaxQuant enables high peptide identification rates, individualized ppb-range mass accuracies and proteome-wide quantification. *Nature Biotechnol* 26:1367-72.

Cox J, Neuhauser N, Michalski A, Scheltema RA, Olsen JV, Mann M (2011). Andromeda: A peptide search engine integrated into the MaxQuant environment. J Proteome Res 10:1794-805.

Feng D, Zhao X, Soromani C, Toikkanen J, Römisch K, Vembar SS, et al (2007). The transmembrane domain is sufficient for Sbh1p function, its association with the Sec61 complex, and interaction with Rtn1p. *J Biol Chem* 282:30618-28.

Geddes JM, Caza M, Croll D, Stoynov N, Foster LJ, Kronstad JW (2016). Analysis of the protein kinase A-regulated proteome of *C. neoformans* identifies a role for the ubiquitin-proteasome pathway in capsule formation. MBio 7:e01862-15.

Gerik KJ, Donlin MJ, Soto CE, Banks AM, Banks IR, Maligie MA, Selitrennikoff CP, Lodge JK (2005). Cell wall integrity is dependent on the *PKC1* signal transduction pathway in *Cryptococcus neoformans*. *Mol Microbiol* 58:393–408.

Liu OW, Chun CD, Chow ED, Chen C, Madhani HD, Noble SM (2008). Systematic genetic analysis of virulence in the fungal pathogen *Cryptococcus neoformans*. Cell 135:174-188.

Rappsilber J, Mann M, Ishihama Y (2007). Protocol for micro-purification, enrichment, pre-fractionation and storage of peptides for proteomics using Stage Tips. Nat Protoc 2:1896-906.

Santiago-Tirado FH, Peng T, Yang M, Hang HC, Doering TL (2015). A single protein S-acyl transferase acts through diverse substrates to determine cryptococcal morphology, stress tolerance, and pathogenic outcome. *PLoS Pathog* 11(5):e1004908.

Skowyra ML, Doering TL (2012). RNA interference in *Cryptococcus neoformans*. *Methods Mol Biol* 845:165–86.

Toikkanen J, Gatti E, Takei K, Saloheimo M, Olkkonen VM, Söderlund H, de Camilli P, Keränen S (1996). Yeast protein translocation complex: Isolation of two genes *SEB1* and *SEB2* encoding proteins homologous to the Sec61ß subunit**.** *Yeast* 12:425-438.

Tyanova S, Temu T, Sinitcyn P, Carlson A, Hein MY, Geiger T, Mann M, Cox J (2016). The Perseus computational platform for comprehensive analysis of proteomics data. Nat Methods 13:731-40.
